# Chronic cerebral hypoperfusion promotes the propagation of tau pathology through AEP‐I_2_^PP2A^ pathway

**DOI:** 10.1002/alz70855_101964

**Published:** 2025-12-23

**Authors:** Xinyi Zhang, Siyan Zhong, Shuai Zhao, Shang Jia, Yaping Yan, Yanxing Chen, Baorong Zhang

**Affiliations:** ^1^ Department of Neurology, the Second Affiliated Hospital, School of Medicine, Zhejiang University, Hangzhou, Zhejiang, China

## Abstract

**Background:**

The pathogenesis of Alzheimer's Disease (AD) remains unknown, which is multifactorial. Neurofibrillary tangles (NFTs) is one of the main pathological changes. Chronic cerebral hypoperfusion (CCH) precedes tau pathology by years and may accelerate its progression. However, the underlying mechanisms remain elusive. Therefore, in the current study, we intended to investigate the effects of CCH on tau propagation.

**Method:**

Tau‐HEK293 cells, stably expressing tau microtubule‐binding repeat domain with yellow fluorescent protein, were transduced with tau PFFs to assess tau aggregation under oxygen‐glucose deprivation (OGD). Tau PFFs were injected into the brains of 2‐ to 3‐month‐old PS19 mice, and unilateral common carotid artery occlusion (UCCAO) was performed to examine the effect of CCH on tau pathology. Western blotting and immunofluorescence staining were used to analyze tau pathology, AEP, I_2_^PP2A^, BBB‐associated proteins, and the activation of microglia in the brain.

**Result:**

We found increased levels of tau aggregates, aspartate endopeptidase (AEP), and I_2_^PP2A^ in OGD treated tau‐HEK293 cells. CCH promoted the propagation of tau pathology in PS19 mice by enhancing the phosphorylation of insoluble tau. Notably, the levels of AEP and I_2_^PP2A^ were increased in the brains of tau PFF‐injected PS19 mice treated with CCH. Additionaly, AEP was found translocate from the lysosome to cytoplasma, and I_2_^PP2A^ translocated from the nucleus to cytoplasma. Moreover, CCH also affected the levels of tight junction proteins, the activation of microglia, and the gene expression profiles in tau PFF‐injected PS19 mice.

**Conclusion:**

These data suggests the involvement of AEP‐I_2_^PP2A^ in enhanced hyperphosphorylation and propagation of tau pathology associated with CCH.

